# A Right Atrial Hemangioma Mimicking Thrombus In A Patient With Atrial Arrhythmias

**DOI:** 10.2174/1874192400701010034

**Published:** 2007-12-31

**Authors:** Alpesh A Patel, Ebere O Chukwu, Daniel S Swerdloff, Vivek Bhatt, Stuart O Schecter, Anastasia Anagnostopoulos, Aasha S Gopal

**Affiliations:** Adult Noninvasive Laboratory, St. Francis Hospital, Roslyn, NY, Stony Brook University, Stony Brook, NY, USA

## Abstract

Cardiac hemangiomas are rare tumors, accounting for only 2.8% of all benign primary cardiac tumors and occur at any age. Clinical presentations vary depending on the tumor location (myocardial, endocardial or pericardial). In many cases, this may be an incidental finding. We report the case of a patient with paroxysmal atrial fibrillation who had a right atrial hemangioma detected with transesophageal echocardiography prior to having percutaneous pulmonary vein isolation performed

## CASE REPORT

**Fig. (1):**
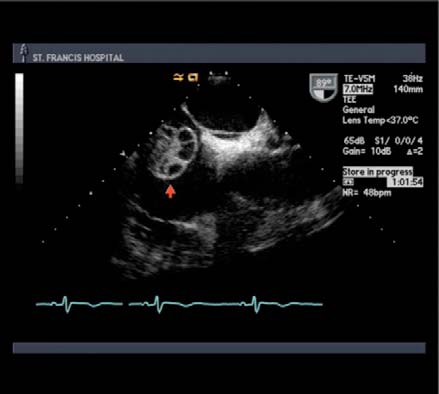
(TEE) Cystic and pedunculated mobile mass with septations measuring approximately 2.5 x 3.5 cm in the right atrium.

**Fig. (2):**
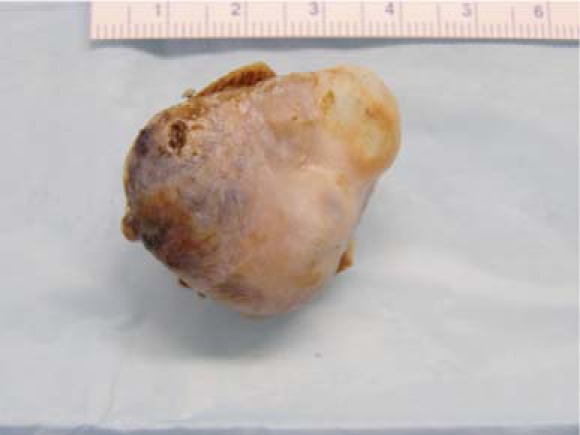
Pedunculated and polypoid gross specimen.

**Fig. (3):**
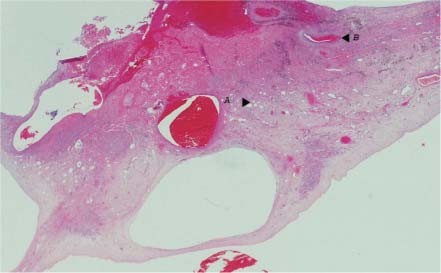
Microsopic section of the specimen depicting focal small capillary channels (**A**) and dilated cavernous vessels (**B**).

A 50-year-old male with history of paroxysmal atrial fibrillation for one year presented for evaluation for pulmonary vein isolation. He had a history of palpitations, but denied syncope, chest pain, or dyspnea. Physical examination was unremarkable except for an irregular heart rate (approximately 90 beats per minute). Computed Tomographic Angiography (CTA) of the pulmonary veins and left atrium was normal. However, a large mass was noted in the right atrium. A presumptive diagnosis of tumor or a large thrombus was made. Transesophageal Echocardiography (TEE) revealed normal cardiac chamber sizes and normal left and right ventricular systolic function. A mobile mass measuri ng approximately 2.5 x 3.5 cm was identified (Fig. **1**). The mass was cystic with septations and was attached to the interatrial septum near the inferior vena cava. Cardiac angiography revealed normal coronaries. The patient underwent open heart surgery to resect the mass and had intra-operative pulmonary vein isolation performed. The mass on gross examination was pedunculated and polypoid (Fig. **2**). On sectioning, the cut surface was hemorrhagic, friable and partially cystic. This led to the preliminary diagnosis of an atrial thrombus. Multiple microscopic sections reveal a fibrotic hemorrhagic stroma with dilated cavernous vessels and focal small capillary channels (Fig. **3**). The mass was covered with endocardium. The final diagnosis was an intracavitary cardiac hemangioma (cavernous and capillary type) of the right atrium. The patient did well and was discharged home in sinus rhythm.

## DISCUSSION

Primary cardiac tumors are far less common than metastatic tumors, with an incidence reported to be approximately 0.02% in a large autopsy series [1]. Most cardiac tumors are benign; however, when found in the right side of the heart, they are more likely to be malignant (75%) than benign (25%) [2]. The most common form of benign cardiac tumor is the myxoma, accounting for 50% of neoplasms, while other forms including rhabdomyomas, hemangiomas, 

lipoma, and fibromas account for the rest. Hemangiomas are very rare, accounting for 2.8% of all benign primary cardiac tumors and usually occur in the left side of the heart making this an unusual case and interesting cause of atrial arrhythmia. They occur in the right side of the heart only in 1 out of 5000 cases. These vascular tumors are made up of a proliferation of endothelial cells, and histologically are similar to hemangiomas in other parts of the body [3]. They are classified histologically into 3 types:

Cavernous: Composed of multiple dilated thin walled vessels.

Capillary: Composed of small capillary like vessels.

Arterio-venous: Composed of dysplastic malformed arteries and veins.

The more commonly encountered forms are the cavernous and capillary types; however, the tumor may have features of all three types [4,5]. The tumor may be located in the myocardium, endocardium or the epicardium. The clinical presentation varies depending on the location and includes arrhythmias, symptoms of heart failure, right ventricular outflow obstruction and cardiac tamponade [5,6,7]. In the absence of right atrial wall invasion, pericardial effusion or tumor originating from the inferior vena cava, a right atrial tumor can be presumed to be benign [8]. However, since statistically, the majority of right sided tumors are malignant, a biopsy and tissue histology are required for a definitive diagnosis. Establishing a diagnosis of cardiac hemangioma can be difficult intraoperatively and indeed initial gross examination of this specimen led to a diagnosis of organizing thrombus given the patient’s history of atrial arrhythmias.

While echocardiography has been used to establish a diagnosis of cardiac hemangioma, there are no standardized criteria [6]. While the cardiac hemangioma is commonly described as a large multiloculated mass with multiple trabeculae and numerous echo free spaces [9], other descriptions can be found in the literature [7]. The most helpful echocardiographic features noted in our case were the recognition of the cystic and septated nature of the mass. Although it was not used in our case, further characterization of the tumor can be made using contrast enhanced echocardiography which can demonstrate the vascularity of the tumors and differentiate it from other relatively avascular structures such as myxomas, lipomas and fibromas [10, 11]. Thus in the assessment of a cardiac mass, use of a combination of echocardiographic features may be helpful in characterizing the nature of the mass. The TEE appearance and location of this mass was inconsistent with the initial pathologic diagnosis of atrial thrombus and led to further staining and the correct characterization of the operative specimen.
